# Housing and health: new evidence using biomarker data

**DOI:** 10.1136/jech-2018-211431

**Published:** 2019-01-14

**Authors:** Amy Clair, Amanda Hughes

**Affiliations:** 1 ESRC Research Centre on Micro-Social Change, Institute for Social and Economic Research, University of Essex, Colchester, UK; 2 MRC Integrative Epidemiology Unit, Population Health Sciences, Bristol Medical School, University of Bristol, Bristol, UK

**Keywords:** housing, health, biomarkers, inflammation, Great Britain

## Abstract

**Background:**

The link between housing and health is well established and long-standing, however much of the evidence relies on self-reported health measures. While these are useful, the availability of biomarker data allows us to add to this evidence using objective indicators of health.

**Methods:**

In this paper, we use C-reactive protein (CRP), a biomarker associated with infection and stress, alongside information relating to housing details, demographic characteristics and health behaviours taken from the UK Household Longitudinal Study. Hierarchical linear regression models estimate CRP for individual housing characteristics, and all available housing characteristics, controlling for confounders.

**Results:**

Results indicate that housing tenure, type, cost burden and desire to stay in current home are associated with CRP. Private renters have significantly higher (worse) CRP than owners with a mortgage. In terms of housing type, respondents living in detached homes had lower CRP than those in semidetached or terraced houses, or those living in flats. Housing cost burden is associated with lower CRP, although further analysis indicates that this is the case only for low-income renters. Desire to stay in current home is significantly associated with higher CRP.

**Conclusions:**

A number of housing characteristics were associated with CRP. These results further support an important role for housing in health.

## Introduction

Concern about the health impacts of housing has a long tradition. Rowntree wrote about the ‘inadequate and insanitary’ housing of the ‘struggling poor’ and the impact of these problems on health in 1901.^1^ In 1934, Britten wrote about the high mortality rates in slum areas of the USA and UK.^2^ His concerns (poor sanitation, overcrowding, poor ventilation and light, disrepair and fire risk) remain relevant to this day. Recent high-profile cases of sanitation problems—such as the contamination of water in Flint, Michigan^3^ and the presence of raw sewage in and around homes in Alabama^4^—and poor fire safety, for example, at Grenfell Tower in London,^5^ have highlighted the importance of continued vigilance to housing issues.

Housing appears to influence health in numerous ways. Recent research has moved beyond the physical characteristics emphasised by Britten, to increasingly recognise the influence of social and psychological housing factors. Shaw^6^ categorised influences of housing on health into ‘hard’ and ‘soft’ types. Hard influences include the physical characteristics of housing that might directly impact health, such as material housing conditions. Soft factors include the meaning of home, and the role a person’s home plays in perceptions of security and social position, including issues around affordability. This categorisation may be thought of as highlighting the dual role of housing as shelter and as home.

Perhaps the most thoroughly studied link between housing and health is that between respiratory health and damp conditions and/or cold indoor temperatures.^6 7^ Damp, and associated mould, are linked with higher levels of dust mites in the home, a cause of asthma.^8^ Damp has also been linked with headaches, diarrhoea, fever and aches and pains,^6^ and more generally has been suggested to ‘create general susceptibility to poor health’.^9^ Cold has been linked with raised blood pressure and cholesterol, as well as excess winter mortality.^10^

In terms of the ‘soft’ influences of housing, a growing body of literature explores associations between health and housing payment difficulties/affordability. A recent paper exploring the impact of a policy change resulting in increased housing spending for those receiving financial support for housing costs found strong evidence of a causal relationship between housing affordability and mental health.^11^ An Australian study suggested poor housing affordability had a greater negative impact on mental health for men than women.^12^

The importance of financial aspects of housing to health is likely due to its role as a key source of ontological security.^6^ Ontological security is ‘the confidence that most human beings have in the continuity of their self-identity and in the constancy of the surrounding social and material environments of action.’^13^ Some have argued home ownership by its nature enables greater feelings of security,^14–16^ and many studies of housing affordability explore tenure differences in the impact of affordability problems. Some evidence suggests the health of homeowners is particularly affected by housing payment problems, more than for renters.^17^

However, tenure differences are context dependent. Work using Australian panel data has found worse health outcomes for renters facing housing payment problems compared with owners,^18^ as did a cross-European study.^19^ Differences in tenure effects may reflect the psychosocial effects of inequalities, where tenure hierarchy and status may vary considerably depending on the context.^20^ Relatedly, they may reflect differences in housing policies. For example, while private renters in the UK have short tenancies and little security compared with owners, in Germany tenancies are indefinite and regulation is more robust.^21^ Thus, health effects may differ.

Tenure is not the only characteristic of housing plausibly impacting ontological security and autonomy. Hiscock *et al*,^16^ in their study of housing in Scotland, argued, ‘house type is of as much importance as tenure in providing autonomy,’ with respondents feeling that flats were limiting, for example, particularly where improvements and maintenance were needed in shared areas. Another Scottish study found people who had moved into a house from a flat reported better mental well-being and quality of life, related to having their own private entrances, which they associated with control and security, as well as access to gardens.^22^

Although extensive evidence links housing with health outcomes, one criticism of existing evidence is that it often relies on self-reported health measures.^6^ While self-reported health is an important health measure, strongly associated with mortality,^23^ there have been calls to complement existing evidence with use of biomarker data.^24^ Biomarkers are ‘objective indications of medical state’ that can be measured ‘accurately and reproducibly’.^25^ In this paper we use a biomarker associated with stress and inflammation and report associations with a range of housing measures.

## Methods

### Data

The UK Household Longitudinal Study (UKHLS) is an annual household panel survey covering approximately 40 000 households in the UK. Beginning in 2009, it replaced the British Household Panel Survey (BHPS), which had been running since 1991, absorbing the BHPS sample in the second wave of the survey. These data include extensive information from individuals and about households, including housing situations. Biological data, including blood samples, were collected at a single, separate nurse visit which occurred between 2010 and 2012.^26^

### Biomarkers

The nurse visit occurred approximately 5 months after the main survey interview, during wave 2 for new UKHLS survey members and wave 3 for BHPS survey members. Biomarker samples were successfully collected for 90.8% of those who were eligible (Survey members who were aged 16 or over, who completed a face-to-face interview, were not pregnant and who completed the survey in English were eligible for the nurse health assessment. For logistical reasons, the nurse health assessment was not conducted in Northern Ireland.^27^) and consented to a blood sample (equivalent to 68.5% of those who participated in the nurse health assessment, 36.5% of all who were eligible for the nurse interview). The resulting sample size is 13 107 people for whom at least one biomarker is available. Further details about eligibility and the sample are available in Benzeval *et al*’s data guidance.^27^

This paper focuses on the biomarker C-reactive protein (CRP), a marker of inflammation associated with infection or stress. CRP levels above 3 mg/L are associated with cardiovascular disease.^27^ CRP is related to unemployment^28^ and low socioeconomic position (SEP),^29^ suggesting CRP is an appropriate biomarker for the study of housing. The association of CRP with both infection and chronic inflammatory processes means CRP elevations could reflect both direct influences of housing on health (eg, low temperature leading to infection) and indirect effects (such as physiological effects of stress associated with unaffordable housing). However, by excluding participants with high CRP (outlined below), we focus principally on indirect pathways relating to chronic processes.

Analysis is limited to cases with valid CRP information (n=11 781 weighted, 12 902 unweighted). Cases with CRP>10 mg/L are removed (n=641 weighted, 716 unweighted) as this is considered an indicator of recent infection.^27^ Those with CRP below the detectable limit of 0.2 mg/L are given a value of 0.1 mg/L. CRP was positively skewed and so log transformed for the regression analysis (see online supplementary appendix figure 1). Analysis is restricted to participants aged over 21 as younger respondents may still be in education (an important predictor of SEP) and living with parents rather than independently. Similarly, a very small number of people aged over 95 are removed. Cases with missing predictor variables are excluded. The final sample size is 9593 (weighted, 9974 unweighted).

10.1136/jech-2018-211431.supp1 Supplementary data

### Housing measures and controls

In order to study the association between housing and health we select indicators of housing situation (table 1), guided by existing evidence from the literature.

**Table 1:** Housing situation measures (weighted, restricted to those valid for all cases)

	n	%
Tenure		
Owned outright	3314	34.55
Owned with mortgage	3682	38.38
Social rent	1484	15.47
Private rent	1114	11.61
Housing payment burden		
Yes	433	4.51
No	9160	95.49
Housing payment arrears		
Yes	679	7.08
No	8914	92.92
Receiving housing benefit		
Yes	1319	13.75
No	8274	86.25
Home has central heating		
Yes	9061	94.45
No	532	5.55
Adequate heating		
Yes	9026	94.09
No	567	5.91
Overcrowding		
Yes	955	9.96
No	8638	90.04
Dwelling type		
Detached	2417	25.2
Semidetached	3101	32.32
Terrace	2738	28.54
Flat	1254	13.07
Other	84	0.87
Prefer to move home		
Yes	3393	35.37
No	6200	64.63
Expect to move in the next 12 months		
Yes	1101	11.48
No	8492	88.52

Housing payment burden: Housing costs account for over one-third of net household income, net household income is below 60% of sample median household income.

Housing payment arrears: In the last 12 months, have you ever found yourself behind with your rent/mortgage?

Adequate heating: In winter, are you able to keep this accommodation warm enough?

Overcrowding: Sufficient bedrooms per person/couple.

We also include a number of variables related to housing characteristics and CRP which could confound associations (table 2). We include age and gender, as CRP levels increase with age and are higher in women than men.^27^ Similarly, tenure varies considerably with age; private renting is the most common tenure among those aged 16–34, whereas in older age groups owner-occupation is more common.^30 31^

**Table 2:** Control variable descriptives (weighted)

	n	%
Gender		
Female	5266	54.90
Male	4327	45.10
Employment status		
Employed	5692	59.34
Unemployed	443	4.61
Retired	2430	25.33
Maternity/caring	573	5.97
Student	101	1.05
Long-term sick/disabled	305	3.18
Other	50	0.52
Ethnicity		
White British	8407	87.64
Other white	434	4.53
Asian	403	4.21
Other	348	3.63
Cohabitation		
Yes	6654	69.36
No	2939	30.64
Region		
North-East	465	4.85
North-West	1084	11.3
Yorkshire and Humber	816	8.51
East Midlands	725	7.55
West Midlands	861	8.98
East of England	937	9.77
London	1192	12.42
South-East	1329	13.86
South-West	849	8.85
Wales	507	5.28
Scotland	828	8.63
Highest qualification		
Degree	2291	23.89
Other higher education (below degree level)	1222	12.74
A-level and similar	1765	18.40
GCSE and similar	1900	19.81
Other qualification	1036	10.80
No qualifications	1378	14.36
Income quartile (equivalised gross household income, standardised by age)		
1	2604	27.14
2	2412	25.15
3	2286	23.83
4	2290	23.88
Current subjective financial situation		
Living comfortably	2751	28.68
Doing alright	3252	33.90
Just about getting by	2555	26.63
Finding it quite difficult	729	7.60
Finding it very difficult	306	3.19
Long-standing illness		
Yes	3430	35.75
No	6163	64.25
Smoking behaviour		
Never smoked	4977	51.88
Ex-smoker	2554	26.62
Current smoker, up to 10 per day	1043	10.88
Current smoker, 11–20 per day	861	8.97
Current smoker, 21+ per day	158	1.64
BMI		
Under 18.5 (underweight)	99	1.03
18.5 to under 25 (recommended weight)	2947	30.72
25 to below 30 (overweight)	3784	39.45
30 to below 40 (class 1 obese)	2508	26.14
40 and above (class 2 obese)	255	2.66

Mean	SD	Min	Max
Age			
50.01	16.84	22	95

BMI, body mass index; GCSE, General Certificate of Secondary Education.

There are differences in housing experiences across ethnicities,^32^ partly due to persistence of discrimination in the housing sector.^33 34^ We therefore include ethnicity, although in broad categories due to small cell sizes for some groups. Cohabitation status is included as it will likely affect the resources available for obtaining housing, as well as the space required. We include Government Office Region because of variation in housing policy between UK countries, and markedly different housing markets across the UK.

We include employment status, by which CRP has been found to differ.^28^ Relatedly, social renters are more likely to be economically inactive or have a low household income than people in other tenures.^31^ Meanwhile, SEP more broadly is associated with both tenure and health, raising the possibility of confounding.^31 35 36^ We therefore also include highest educational qualification and an indicator of income. (We include income quartile equivalised by age, where income reflects gross household income in the month before survey response, equivalised using the modified Organisation for Economic Co-operation and Development equivalence scale, adjusted for inflation. Inflation data from https://data.oecd.org/price/inflation-cpi.htm.) Alongside income, we include a variable reflecting subjective financial situation, to capture financial strain.

Smoking status and body mass index (BMI) are included because of the established relationships between smoking, adiposity and increased inflammation,^37 38^ as well as the uneven distribution of these characteristics across tenures, likely reflecting otherwise unmeasured social characteristics as well as selection into tenure. Smoking variables are only available at wave 2, so these values are also used for people who had biomarker collection at wave 3. BMI (kg/m^2^) is calculated from height and weight measured by a trained nurse using a Frankfort plane and digital floor scales, and categorised using standard WHO classifications (http://www.euro.who.int/en/health-topics/disease-prevention/nutrition/a-healthy-lifestyle/body-mass-index-bmi). Since raised inflammation may reflect pre-existing chronic illness, a binary self-report indicator of long-standing illness is included.

### Analysis

We first explore mean levels of CRP for people in different housing situations, controlling for age and gender. Following this, we run a series of linear regressions in Stata, accounting for survey design. Initial models explore associations of individual housing characteristics with CRP (model 1). Controls are then added for demographic characteristics and SEP (model 2), and following this for health and health behaviours (model 3). The final model includes all selected housing characteristics, as well as controls for demographic characteristics, SEP, health and health behaviours (model 4). All models apply inverse-probability weights to address differential sampling and response probabilities.

## Results

### Preliminary results

Within our sample mean CRP is 2.01 (untransformed), with an SD of 1.98, minimum 0.1 and maximum of 9.9. 22.08% of participants have ‘raised’ (above 3 mg/L but below 10 mg/L) CRP (weighted).

**Figure 1:**
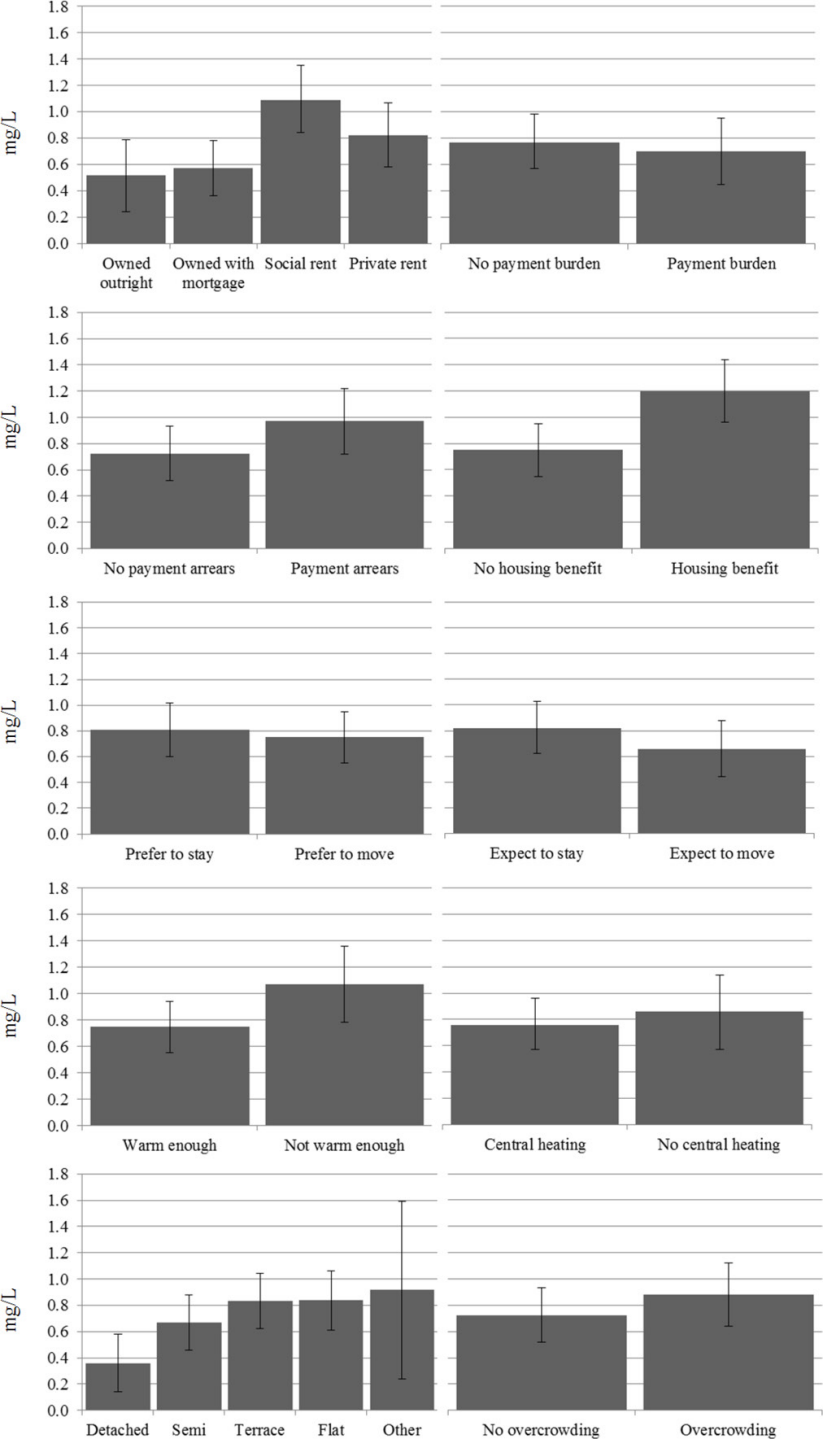
Mean C-reactive protein (CRP) controlling for age and gender.

We first report associations between the housing variables and inflammatory markers, controlling for age and gender (figure 1). Few statistically significant differences are found. For tenure, social renters have the highest CRP. Given the nature of selection into this tenure this should not be surprising, however CIs overlap with those for private renters. For the financial variables, those receiving housing benefit have higher CRP than those not receiving housing benefit. For building type, CRP is considerably lower for those living in detached homes than other categories. The CIs overlap with those for semidetached homes but not for terraces or flats.

### Multivariate results

Results of regression analyses (table 3) indicate that, with all other housing variables controlled for, private renters have significantly higher CRP than those who own their home with a mortgage. Accounting for all other housing variables, housing type is still predictive of CRP, with higher CRP for those living in semidetached and terraced houses, or those living in flats, compared with those in detached houses. Those who want to stay in their own home have slightly higher CRP levels.

**Table 3:** Linear regression models predicting log of C-reactive protein

	B SE	B SE	B SE	B SE
	Model 1	Model 2	Model 3	Model 4
Housing tenure (ref: owned with mortgage)				
Owned outright	0.22***	−0.02	0.01	0.02
	0.03	0.03	0.03	0.03
Social rent	0.36***	0.18***	0.09*	0.08
	0.04	0.04	0.04	0.05
Private rent	0.06	0.12*	0.11*	0.10*
	0.05	0.05	0.05	0.05
Housing cost burden (yes)	−0.10	−0.15*	−0.10	−0.14*
	0.07	0.07	0.07	0.07
Housing payment arrears (yes)	0.05	0.06	0.01	0.00
	0.06	0.06	0.05	0.05
Housing benefit (yes)	0.26***	0.09	0.01	−0.05
	0.04	0.05	0.04	0.05
Central heating (no)	0.07	0.02	0.04	0.04
	0.05	0.05	0.05	0.05
Warm enough (no)	0.13*	0.02	−0.03	−0.04
	0.06	0.06	0.06	0.06
Overcrowding (yes)	−0.03	0.05	0.01	0.00
	0.05	0.06	0.05	0.05
Dwelling type (ref: detached)				
Semidetached	0.10***	0.11***	0.06*	0.07*
	0.03	0.03	0.03	0.03
Terrace	0.15***	0.19***	0.13***	0.13***
	0.03	0.03	0.03	0.03
Flat	0.11*	0.20***	0.18***	0.18***
	0.05	0.05	0.04	0.05
Other	0.26	0.21	0.17	0.16
	0.16	0.17	0.17	0.17
Prefer to stay	0.12***	0.04	0.06*	0.05*
	0.03	0.03	0.03	0.03
Expect to move	−0.20***	−0.06	−0.03	−0.06
	0.04	0.05	0.04	0.05
Prefer to stay × expect to move	–	–	–	0.15
				0.10
Demographics and SEP controls included	N	Y	Y	Y
Health and health behaviour controls included	N	N	Y	Y

Demographic and socioeconomic position controls: age, gender, cohabitation, ethnicity, region, employment status, highest educational qualification, income quartile (age standardised), subjective financial situation.

Health and health behaviour controls: smoking status, body mass index (categorised), long-standing illness or disability.

Regressions for housing characteristics run separately in models 1–3, but presented in a single column for ease of comparison and conciseness. All housing variables are included simultaneously in model 4.

n=9974.

*P<0.05; **P<0.01; ***P<0.005.

Unexpectedly, experiencing housing payment burden (spending more than one-third of household income on housing costs, while income is below 60% of the median) is associated with lower CRP. We investigate whether this may be due to a protective effect for higher housing spending for low-income renters. Mean CRP levels are higher for renters (both social and private) who are not experiencing housing burden compared with renters experiencing burden, while the reverse is true for owners with a mortgage. (Outright owners have no housing costs and so none experience housing cost burden. Social renters however may experience housing cost burden as social housing rents in the UK are not limited according to household income.) A regression model interacting tenure with housing cost burden (table 4) confirms this difference, finding higher CRP levels for private renters, but lower levels of CRP among renters experiencing housing cost burden.

**Table 4:** Linear regression model predicting log of C-reactive protein, interaction model

	B SE
Housing tenure (ref: owned with mortgage)	
Owned outright	0.03
	0.03
Social rent	0.09
	0.05
Private rent	0.14**
	0.05
Housing payment burden (yes)	0.12
	0.10
Housing payment burden × social rent	−0.28*
	0.13
Housing payment burden × private rent	−0.45**
	0.15

Full model equivalent to model 4 in table 3, including the following covariates: age, gender, cohabitation, ethnicity, region, employment status, highest educational qualification, income quartile (age standardised), subjective financial situation, smoking status, body mass index (categorised), long-standing illness or disability. Limited results shown here.*P<0.05; **P<0.01; ***P<0.005.

## Robustness checks

To check robustness of findings, we run a number of additional analyses. Models are run excluding participants taking statins (n=1503) or anti-inflammatory (n=538) medication, which influence CRP levels. This does not substantively affect results (online supplementary appendix table 1).

10.1136/jech-2018-211431.supp2 Supplementary data

To check timing of collection or processing of biomarkers does not affect the results, we run a model including the wave at which biomarker information was collected (83.67% responded at wave 2; 16.33% at wave 3, weighted). A second includes the month of data collection, given plausible seasonal variation in illness and stress. Neither addition substantively affects results (online supplementary appendix table 1).

A model accounting for noise from neighbours is presented to check if the significant finding for housing type is due to lower noise levels in detached homes. As noise was only included in wave 3 of the survey, we use the noise response from wave 3 for all participants, and rerun models including noise and interactions between noise and housing type. Even with these adjustments, housing type significantly predicts CRP (online supplementary appendix table 2).

We include indicators of BMI and smoking status in our analysis. Given that a degree of mediation between these indicators and housing characteristics is plausible, estimates are likely to be conservative. We report a version of our final model (model 4, table 3) without these controls in the appendix (online supplementary appendix table 3). Results are broadly similar, with the exception being that in the model excluding BMI and smoking living in social housing is associated with higher CRP levels. This likely reflects compositional differences, 32% of the social rent respondents reported having never smoked, compared with over 57% of owners (both outright and with a mortgage), for example.

## Discussion

This analysis investigates the association between people’s housing experiences and their health, measured using CRP. Results indicate that living in the private rented sector, a home other than one that is detached, or wanting to stay in the current home is associated with higher CRP.

That housing type is associated with CRP provides support for Hiscock *et al*’s^16^ finding that housing type is important for health. It may be that access to gardens/green space, which likely varies according to housing type, may partially explain this result, but this could not be tested with the current data. The significant findings for housing type and tenure point to an influence of autonomy and control. Where control is low, ontological security is reduced, which may affect health through chronic stress responses.^39^

For a number of variables where a relationship with health was expected—the home being warm enough, expecting to move, anticipating a forced move—no statistically significant association with CRP is found. The lack of a significant finding for expecting to move may be due to the positive and negative reasons for moves cancelling each other out in the analysis. The number of people anticipating forced moves was small, affecting less than 3% of the sample. Thus, although the point estimate for CRP is consistent with a greater elevation for this group than for the elevation associated with living in a flat, the lack of statistical significance is perhaps not surprising. While for the thermal comfort variable, research has found a complex relationship between temperature and health (eg, Sutton-Klein *et al* 40), the lack of a statistically significant finding likely reflecting this complexity.

Unexpectedly, facing housing payment burden is associated with lower CRP levels. We hypothesise that this could be because higher expenditure on housing enables people in lower income households to access better quality rented housing, the health benefits of this outweighing the damaging effects of financial strain. Further research, using surveys which collect information on factors such as damp and mould, will be required to test this hypothesis.

### Limitations

A limitation of this analysis is that the UKHLS does not contain information on housing conditions such as damp or mould, particularly relevant to the hypothesis that high housing cost burden among renters may positively influence CRP through reduced exposure to poor housing conditions.

The approximately 5-month gap between the main interview and biomarker collection means that housing situations at biomarker collection may differ from those recorded, potentially affecting results. Biomarker data were not collected for children, so our analysis considers only adult health.

The use of household survey data means that we do not consider homelessness, despite obvious health links. Perhaps most importantly, the data used here are cross-sectional, as biomarkers have only been collected once in UKHLS. This precludes longitudinal investigation of causal relationships between housing and CRP, and instead we report associations.

## Conclusions

This paper identifies a number of ways in which housing situations are associated with adult CRP levels. Higher CRP, indicating worse health, is found among those living in the private rented sector. This finding supports arguments for greater consideration of the negative effects of the current private rented market in the UK, characterised by greater insecurity, higher cost and lower quality than is typically found in other tenures. However, due to the cross-sectional nature of the data, this paper cannot make causal claims. Further work will be required to investigate whether specific aspects of the private rented sector, such as housing quality, are responsible for the link between private renting and CRP. Results also find lower CRP levels among those living in detached houses, even after accounting for different aspects of SEP. This points to a possible role of autonomy, which may be greater for people living in detached houses than in other types of buildings. Overall, results of this analysis using biomarker data complement existing work using self-report measures, providing further evidence of an important association between housing and health.

**What is already known on this subject**

- A range of research, both qualitative and quantitative, has found associations between housing experiences and health outcomes.
- Much of this evidence has used self-reported measures of health.

**What this study adds**

- This paper uses C-reactive protein to build on this research by exploring the association between housing experiences and an objective measure of health.
- The analysis finds that C-reactive protein is associated with tenure, building type, housing cost burden and desire to stay in current home.
- These results support arguments that health outcomes should be a consideration of housing policies.
