# Supplementary figures and images for: Housing and health: new evidence using biomarker data
Source: J Epidemiol Community Health. 2019 Jan 14;73(3):256–62. doi: 10.1136/jech-2018-211431 (PMC6580751; doi:10.1136/jech-2018-211431)

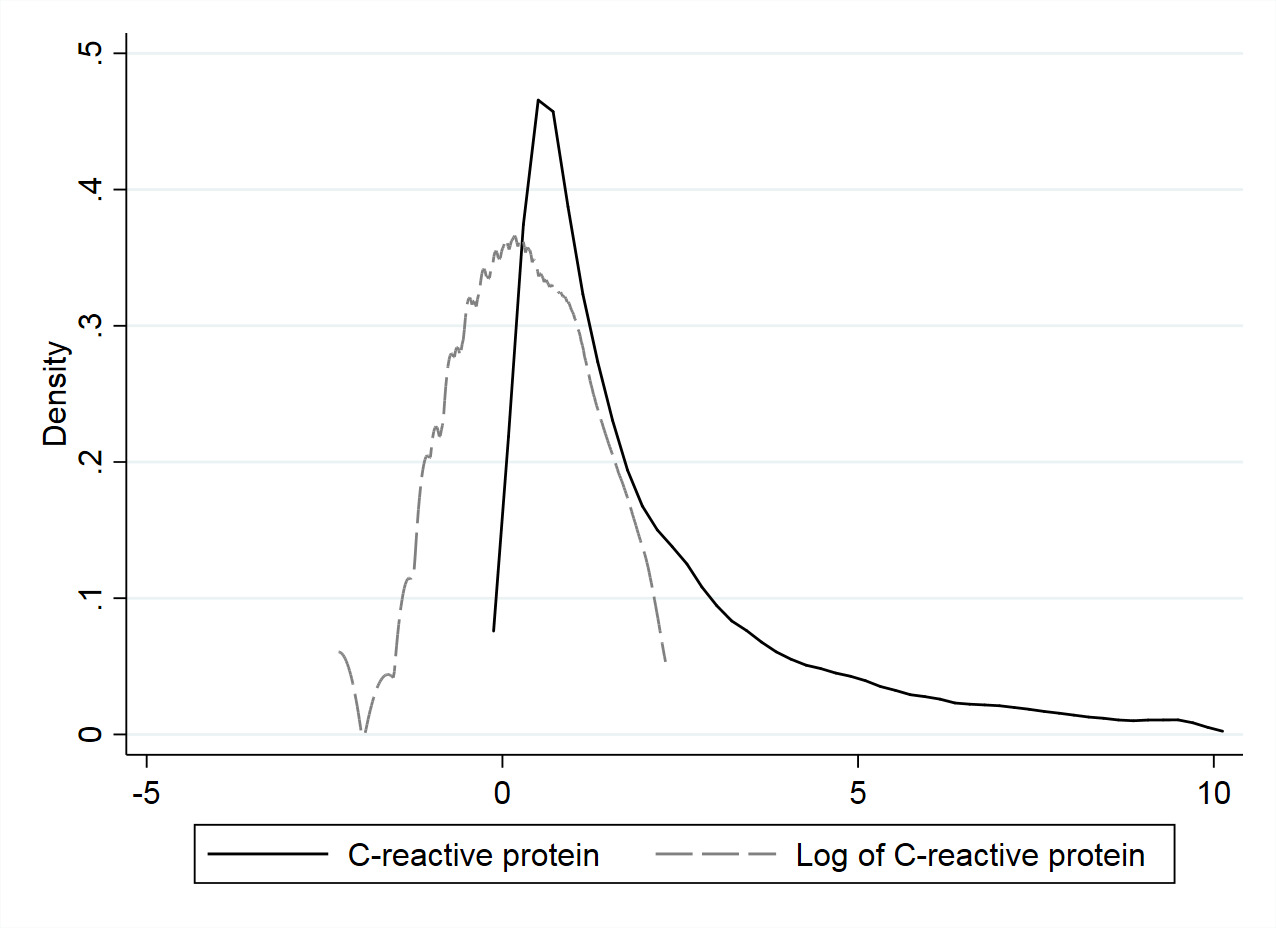

Supplement: Supplementary data [file jech-2018-211431supp001.jpg]
