# Supplementary material for: Housing and health: new evidence using biomarker data
Source: J Epidemiol Community Health. 2019 Jan 14;73(3):256–62. doi: 10.1136/jech-2018-211431 (PMC6580751; doi:10.1136/jech-2018-211431)
Supplement: Supplementary data [file jech-2018-211431supp002.pdf]

Appendix Table 1: Robustness check models

	1. Original	2. Exc. Med	3. Wave	4. Month
**Housing tenure (ref. Owned with mortgage)**				
Owned outright	0.02<br>(0.03)	0.03<br>(0.04)	0.02<br>(0.03)	0.02<br>(0.03)
Social rent	0.08<br>(0.05)	0.05<br>(0.05)	0.07<br>(0.05)	0.08<br>(0.05)
Private rent	0.10*<br>(0.05)	0.11*<br>(0.05)	0.10*<br>(0.05)	0.10*<br>(0.05)
**Housing payment burden (yes)**	-0.14*<br>(0.07)	-0.17*<br>(0.08)	-0.14*<br>(0.07)	-0.14*<br>(0.07)
**Housing payment difficulties (yes)**	0.00<br>(0.05)	0.03<br>(0.06)	-0.00<br>(0.05)	-0.00<br>(0.05)
**Housing benefit (yes)**	-0.05<br>(0.05)	-0.02<br>(0.06)	-0.05<br>(0.05)	-0.05<br>(0.05)
**Central heating (no)**	0.04<br>(0.05)	0.04<br>(0.05)	0.04<br>(0.05)	0.04<br>(0.05)
**Warm enough (no)**	-0.04<br>(0.06)	-0.04<br>(0.07)	-0.04<br>(0.06)	-0.04<br>(0.06)
**Overcrowding (yes)**	0.00<br>(0.05)	-0.01<br>(0.05)	0.00<br>(0.05)	0.01<br>(0.05)
**Dwelling type (ref. Detached)**				
Semi detached	0.07*<br>(0.03)	0.07*<br>(0.03)	0.07*<br>(0.03)	0.06*<br>(0.03)
Terrace	0.13***<br>(0.03)	0.15***<br>(0.04)	0.13***<br>(0.03)	0.13***<br>(0.03)
Flat	0.18***<br>(0.05)	0.17**<br>(0.05)	0.18***<br>(0.05)	0.18***<br>(0.05)
Other	0.16<br>(0.17)	0.23<br>(0.23)	0.17<br>(0.17)	0.17<br>(0.17)
**Prefer to stay**	0.05*<br>(0.03)	0.04<br>(0.03)	0.05*<br>(0.03)	0.05*<br>(0.03)
**Expect to move**	-0.06<br>(0.05)	-0.07<br>(0.06)	-0.06<br>(0.05)	-0.06<br>(0.05)
**Prefer to stay x expect to move**	0.15<br>(0.10)	0.21<br>(0.11)	0.15<br>(0.10)	0.14<br>(0.10)
Constant	-1.12***<br>(0.09)	-1.17***<br>(0.10)	-1.12***<br>(0.09)	-1.15***<br>(0.10)
Observations	9,974	7,814	9,974	9,974
R-squared	0.20	0.22	0.20	0.20

Notes: All models include the following controls: demographic and socio-economic position controls – age, gender, cohabitation, ethnicity, region, employment status, highest educational qualification, income quartile (age standardised), subjective financial situation. Health and health behaviour controls – smoking status, body mass index (categorised), longstanding illness or disability..
Column 2 excludes all respondents taking statins or anti-inflammatories. Column 3 includes the wave in which the biomarker data was collected in addition to the above variables. Column 4 includes the month of biomarker collection in addition to the above variables.

Appendix Table 2: Robustness check models

	1. Original	2. Noise	3. Noise x Housing type
**Dwelling type (ref. Detached)**			
Semi detached	0.06*<br>(0.03)	0.07*<br>(0.03)	0.06*<br>(0.03)
Terrace	0.13***<br>(0.03)	0.15***<br>(0.03)	0.14***<br>(0.03)
Flat	0.18***<br>(0.04)	0.20***<br>(0.04)	0.22***<br>(0.05)
Other	0.17<br>(0.17)	0.18<br>(0.17)	0.21<br>(0.21)
**Noise (yes)**	-	-0.09**<br>(0.03)	-0.13<br>(0.07)
**Noise x semi detached**	-	-	0.08<br>(0.09)
**Noise x terrace**	-	-	0.03<br>(0.09)
**Noise x flat**	-	-	-0.02<br>(0.10)
**Noise x other**	-	-	-0.11<br>(0.27)
Constant	-1.09***<br>(0.09)	-1.07***<br>(0.09)	-1.06***<br>(0.09)
Observations	9,974	9,974	9,974
R-squared	0.20	0.20	0.20

Notes: All models include the following controls: demographic and socio-economic position controls – age, gender, cohabitation, ethnicity, region, employment status, highest educational qualification, income quartile (age standardised), subjective financial situation. Health and health behaviour controls – smoking status, body mass index (categorised), longstanding illness or disability.

Appendix Table 3: Full models with and without BMI and Smoking indicators

	1. Original	2. Without BMI and Smoking
**Housing tenure (ref. Owned with mortgage)**		
Owned outright	0.02<br>(0.03)	-0.01<br>(0.04)
Social rent	0.08<br>(0.05)	0.14**<br>(0.05)
Private rent	0.10*<br>(0.05)	0.12*<br>(0.05)
**Housing payment burden (yes)**	-0.14*<br>(0.07)	-0.18*<br>(0.07)
**Housing payment difficulties (yes)**	0.00<br>(0.05)	0.03<br>(0.06)
**Housing benefit (yes)**	-0.05<br>(0.05)	-0.02<br>(0.05)
**Central heating (no)**	0.04<br>(0.05)	0.02<br>(0.05)
**Warm enough (no)**	-0.04<br>(0.06)	-0.01<br>(0.06)
**Overcrowding (yes)**	0.00<br>(0.05)	0.03<br>(0.06)
**Dwelling type (ref. Detached)**		
Semi detached	0.07*<br>(0.03)	0.10***<br>(0.03)
Terrace	0.13***<br>(0.03)	0.16***<br>(0.03)
Flat	0.18***<br>(0.05)	0.16***<br>(0.05)
Other	0.16<br>(0.17)	0.19<br>(0.17)
**Prefer to stay**	0.05*<br>(0.03)	0.04<br>(0.03)
**Expect to move**	-0.06<br>(0.05)	-0.11<br>(0.06)
**Prefer to stay x expect to move**	0.15<br>(0.10)	0.14<br>(0.11)
Constant	-1.12***<br>(0.09)	-0.79***<br>(0.10)
Observations	9,974	9,974
R-squared	0.20	0.07

Notes: Column 1 is Model 4 from Table 3, Column 2 reports the same analysis excluding BMI and smoking status.
